# Identification of Novel Regulatory Genes in Development of the Avian Reproductive Tracts

**DOI:** 10.1371/journal.pone.0096175

**Published:** 2014-04-24

**Authors:** Whasun Lim, Gwonhwa Song

**Affiliations:** Department of Biotechnology, College of Life Sciences and Biotechnology, Korea University, Seoul, Republic of Korea; Cardiff University, United Kingdom

## Abstract

The chicken reproductive system is unique in maintaining its functions including production of eggs or sperm, fertilization of the egg by sperm maintained in sperm nests, production of hormones regulating its growth, development and function, and reproduction. Development of the reproductive organs is a highly regulated process that results in differentiation and proliferation of germ cells in response to predominant regulatory factors such as hormones and transcription factors. However, only a few genes are known to determine morphogenesis of the chicken reproductive tract and their mechanisms are unknown. Therefore, in the present study, we investigated the expression patterns of four genes including SNCA, TOM1L1, TTR and ZEB1 in the gonads at embryonic days 14 and 18, and in immature (12-week-old) and mature (50-week-old) chickens, as well as the reproductive tract including ovary, oviduct and testes of the respective sexes by qRT-PCR, *in situ* hybridization and immunofluorescence analyses. The expression of SNCA, TOM1L1 and ZEB1 genes was higher in immature and mature female reproductive tracts than expression of TTR. In addition, different temporal and spatial patterns of expression of the four genes were observed during maturation of testis in chickens. Specifically, SNCA, TOM1L1 and TTR were highly expressed in testes of 12-week-old chickens. Moreover, several chicken specific microRNAs (miRs) were demonstrated to affect expression of target gene mRNAs by directly binding to the 3′-UTR of their target genes through actions at the post-transcriptional level as follows: *miR-153* and *miR-1643* for SNCA; miR-1680* for TTR; and *miR-200b* and *miR-1786* for ZEB1. These results suggest that four-selected genes play an important role in development of the male and female reproductive tract in chickens and expression of most candidate genes is regulated at the post-transcriptional level through specific microRNAs.

## Introduction

The chicken is an invaluable animal model for research on embryology and reproductive developmental biology. In avian models, sexual differentiation of the reproductive system is initiated in the embryonic gonads from embryonic day 6.5 (E6.5, HH30) to be either ovaries or testes and this occurs asymmetrically in females and symmetrically in males. It depends on which component of the embryonic gonad, cortex or medullary, is colonized by germ cells that migrate there through bloodstream [1], [2], [3]. In the female (ZW), germ cells asymmetrically populate the left and right gonads. The thickened outer cortex only develops in the left gonad while medulla cords form lacunae. However, the right gonad regresses and fails to develop the cortex. Female germ cells enter meiosis that commences between E14 and E18 in the left gonad [4], [5], [6]. Then, by beginning folliculogenesis, granulosa and theca cells surrounding the oocyte are formed from cells in the cortex of the left functional gonad. There are several candidate genes, forkhead box L2 (FOXL2), wingless-related MMTV integration site 4 (WNT4), proprotein convertase subtilisin/kexin type 6 (PCSK6) and bone morphogenetic protein 6 (BMP6) known to regulate follicular development [3], [7], [8].

The chicken oviduct has a major role in that it is a reproductive tract that produces eggs. The oviduct derives from the Mullerian duct that develops only on the left side in female chicks whereas the right Mullerian duct degenerates in female chicks and both of the Mullerian ducts degenerate in male chicks. The immature oviduct develops rapidly after 16 weeks of age and egg laying begins when chickens are 16- to 20-weeks-old [9], [10]. The oviduct of laying hens consists of four specific segments which are the infundibulum (fertilization), magnum (production of egg-white proteins), isthmus (formation of the soft shell membrane) and shell gland (formation of the outer egg shell) [11]. Development of the oviduct is stimulated in response to estrogen and previous studies have shown the regulatory genes, serpin peptidase inhibitor, clade B (ovalbumin), member 3 (SERPINB3) [12], SERPINB11 [13], adenosylhomocysteinase-like 1 (AHCYL1) [14] alpha 2 macroglobulin (A2M) [15] and pleiotrophin (PTN) [16] are highly expressed during development of the immature oviduct in chickens.

In contrast to female reproductive organs, gonadal morphogenesis leading to a mature testis is symmetrical between left and right gonads and originates in thickened medullary cords via proliferation of Sertoli cells within the cords that are anlage of the seminiferous tubules of male embryos (ZZ). Testes of 6-week-old chicken have seminiferous tubules that include a simple layer of spermatogonia, Sertoli cells, basal lamina and myoid cells. In testes of 50-week-old chickens all stage of spermatogenesis from spermatogonia to spermatozoa are found along with Sertoli cells surrounded by basal lamina and myoid cells [3], [17]. In development of testis, testes-determining genes such as doublesex and mab-3 related transcription factor 1 (DMRT1) (Z-linked gene) and sex determining region Y-box 9 (SOX9) (sertoli cell differentiation factor) participate in testicular morphogenesis [18]. However, cell- and tissue-specific regulation for spermatogenesis remains unknown.

In a previous study, we identified several novel genes based on significant changes in their expression and functional categorization of genes changed between left and right gonads at embryonic days 6 and 9 through microarray analysis that may regulate gonadal morphogenesis in the both sexes of chicken embryos. We focused on four genes including synuclein alpha (SNCA), target of myb 1 (chicken) like 1 (TOM1L1), transthyretin (TTR) and zinc finger E-box binding homeobox 1 (ZEB1) that are associated with cellular proliferation and embryonic development and regulated by FSH and LH for E6 gonadal cells. However, these genes have not been investigated with respect to their influence on development of chicken reproductive tract. Therefore, we hypothesized that these selected genes effect changes in morphogenesis of reproductive organs in chickens. Accordingly, we determined differential patterns of mRNA expression and verified cell- and tissue-specific localization of mRNAs and proteins encoded by the four genes of interesting during development of female and male reproductive tracts in chickens. Moreover, we investigated post-transcriptional regulation of expression of three of the genes (SNCA, TTR and ZEB1) using a miRNA target validation assay. Results of present study provide novel insights into SNCA, TOM1L1, TTR and ZEB1 genes with respect to their tissue-specific expression during differentiation of germ cells into mature reproductive organs and post-transcriptional regulation of their expression by specific miRNAs in chickens.

## Results

### Comparative Expression of SNCA during Development of Reproductive Organs in both Sexes of Chickens

**Figure 1:**
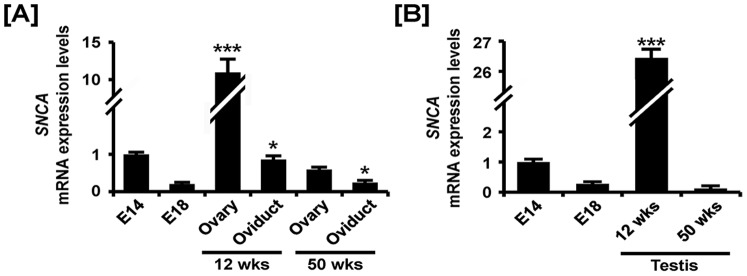
Quantitative analysis of *SNCA* mRNA expression in female and male reproductive tracts during their development. Quantitative RT-PCR was conducted using cDNA templates from female (A) and male (B) gonads at embryonic days 14 and 18, 12-week-old ovary and oviduct and 50-week-old ovary and oviduct. The asterisks denote statistically significant differences (*** *p*<0.001 and * *p*<0.05).

**Figure 2:**
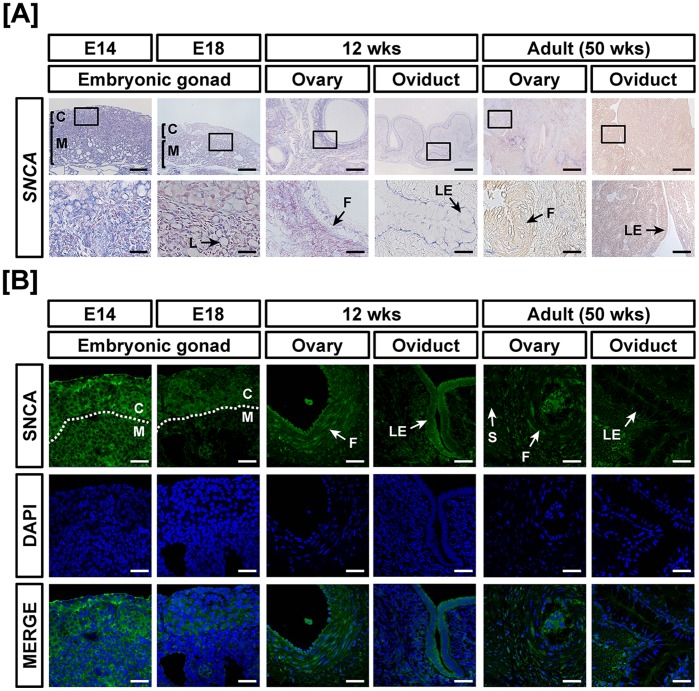
Cell-specific localization of mRNA and protein for SNCA in female reproductive tracts during their development. Cell-specific expression of *SNCA* mRNA and protein in development of the female reproductive tract was demonstrated by *in situ* hybridization (A) and immunofluorescence analyses (B). Cell nuclei were stained with DAPI (blue). Legend: C, cortex; F, follicle; L, lacunae; LE, luminal epithelium; M, medullar. Scale bar represents 100 µm and 20 µm for first and second horizontal panels of (A) and 50 µm for (B). See *Materials and Methods* for a complete description of the methods.

**Figure 3:**
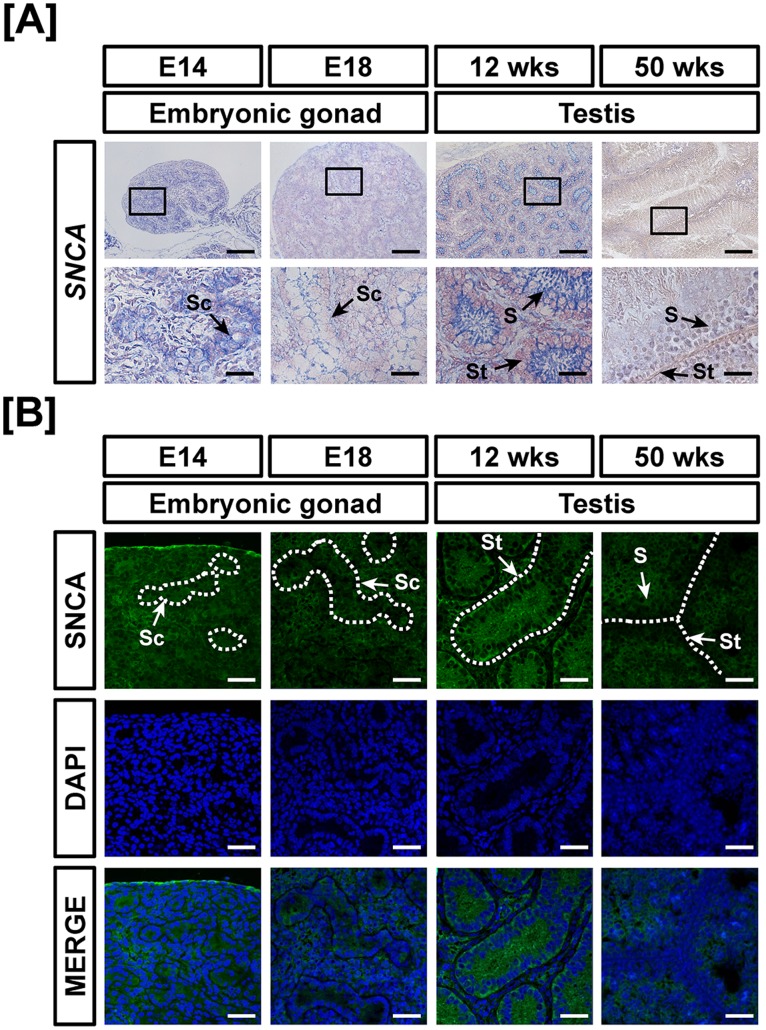
Cell-specific localization of mRNA and protein for SNCA in male reproductive tracts during their development. Localization of SNCA expression was analyzed in the male reproductive tract of chickens during their development by *in situ* hybridization (A) and immunofluorescence analyses (B). Cell nuclei were stained with DAPI (blue). Legend: S, Sertoli cell; Sc, seminiferous cord; St, seminiferous tubule. Scale bar represents 100 µm and 20 µm for first and second horizontal panels of (A) and 50 µm for (B). See *Materials and Methods* for a complete description of the methods.

As illustrated in Figure 1A, the results from quantitative RT-PCR analyses indicated that expression of *SNCA* mRNA decreased 80% in left gonads at E18, 20% (*P*<0.05) in oviducts of 12 week old chicks and 40% and 76% (*P*<0.05) in the adult ovary and oviduct at 50 weeks, respectively as compared with *SNCA* expression in the gonads at E14. On the other hand, *SNCA* mRNA expression increased 11.2-fold (*P*<0.001) in the ovaries of 12-week-old chickens. Moreover, expression of *SNCA* decreased 70% and 90% in the gonads at E18 and testis of 50-week-old chickens, respectively as compared with *SNCA* expression in the gonads at E14 (Figure 1B). However, *SNCA* increased 26.3-fold (*P*<0.001) in the testis of 12-week-old chickens as compared with expression of *SNCA* in E14 gonads. *In situ* hybridization and immunofluorescence analyses detected *SNCA* mRNA and protein localized mainly in the cortex region of embryonic gonads and both were highly expressed in ovarian follicles of the immature chicken oviduct (Figure 2A and 2B). In males, in accordance with mRNA expression, SNCA protein was localized to the seminiferous cord of gonads at E14 and abundance decreased to E18. Interestingly, SNCA protein was abundant in the seminiferous tubules of 12-week-old testis and weakly expressed in Sertoli cells of 50-week-old testes (Figure 3A and 3B).

### Comparative Expression of TOM1L1 during Development of Reproductive Organs in both Sexes of Chickens

**Figure 4:**
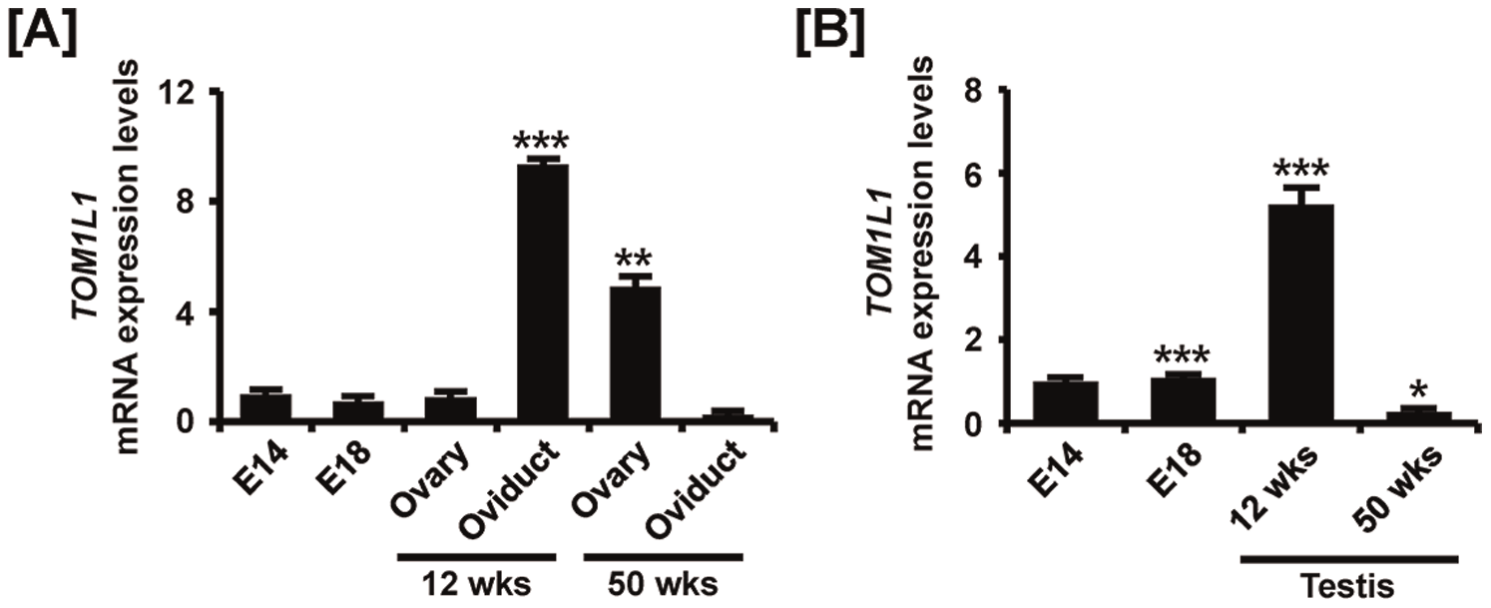
Quantitative analysis of *TOM1L1* mRNA expression in female and male reproductive tracts during their development. Quantitative RT-PCR was conducted using cDNA templates from female (A) and male (B) gonads at embryonic days 14 and 18, 12-week-old ovary and oviduct and 50-week-old ovary and oviduct. The asterisks denote statistically significant differences (*** *p*<0.001, ** *p*<0.01 and * *p*<0.05).

**Figure 5:**
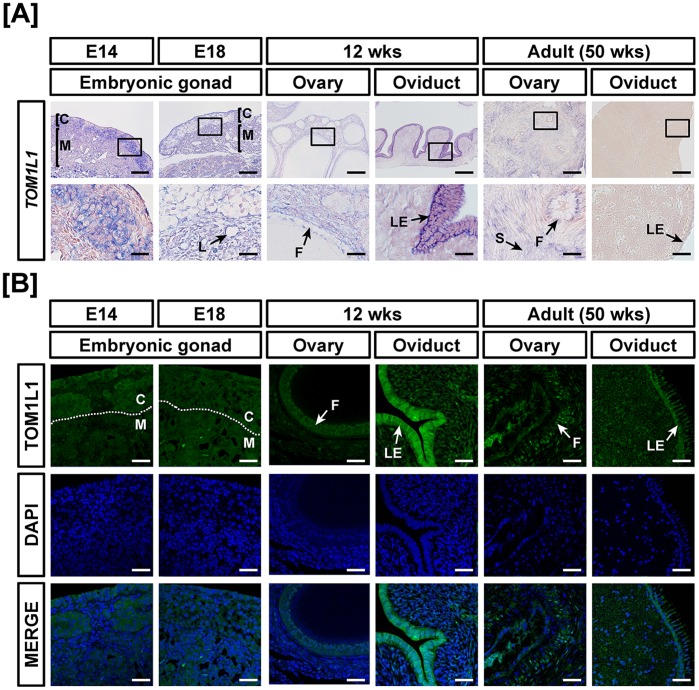
Cell-specific localization of mRNA and protein for TOM1L1 in female reproductive tracts during their development. Cell-specific expression of *TOM1L1* mRNA and protein in development of the female reproductive tract was demonstrated by *in situ* hybridization (A) and immunofluorescence analyses (B). Cell nuclei were stained with DAPI (blue). Legend: C, cortex; F, follicle; L, lacunae; LE, luminal epithelium; M, medullar. Scale bar represents 100 µm and 20 µm for first and second horizontal panels of (A) and 50 µm for (B). See *Materials and Methods* for a complete description of the methods.

**Figure 6:**
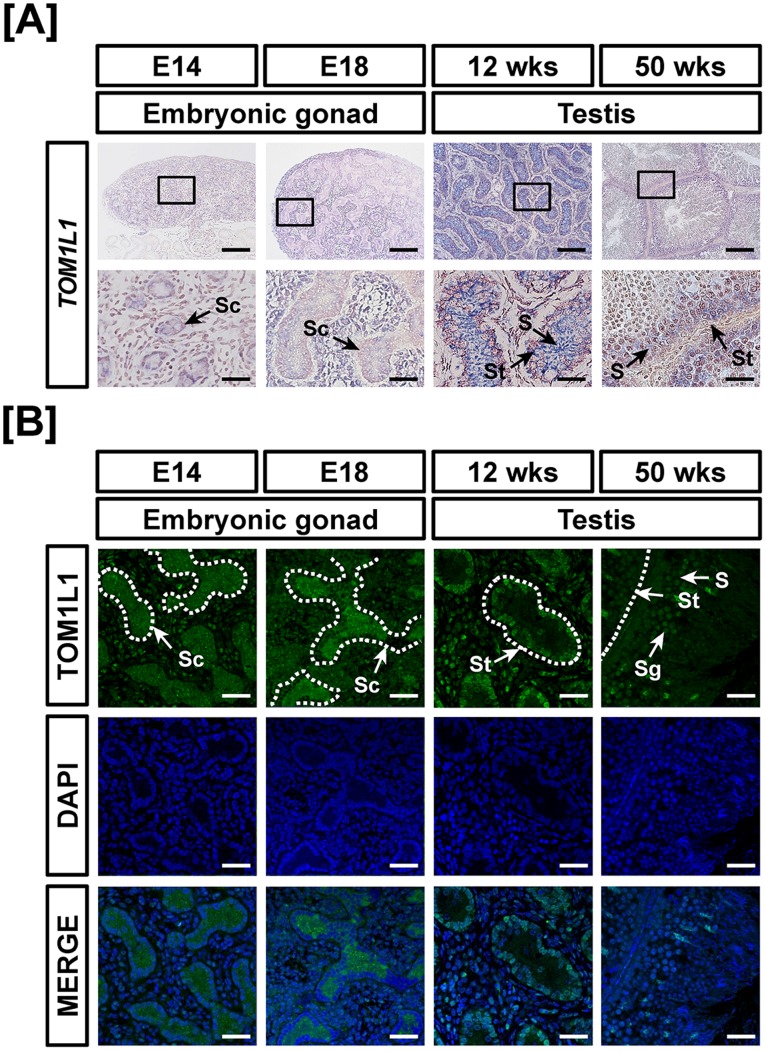
Cell-specific localization of mRNA and protein for TOM1L1 in male reproductive tracts during their development. Localization of TOM1L1 expression was analyzed in the male reproductive tract of chickens during their development by *in situ* hybridization (A) and immunofluorescence analyses (B). Cell nuclei were stained with DAPI (blue). Legend: S, Sertoli cell; Sc, seminiferous cord; Sg, spermatogonia; St, seminiferous tubule. Scale bar represents 100 µm and 20 µm for first and second horizontal panels of (A) and 50 µm for (B). See *Materials and Methods* for a complete description of the methods.

Tissue specific expression of *TOM1L1* mRNA was found to increase 9.4- (*P*<0.001) and 4.9- (*P*<0.01) fold in oviducts at 12-weeks and ovaries at 50 weeks, respectivelyand decrease 70% in oviducts at 50 weeks as compared with *TOM1L1* expression in the female gonads at E14 by quantitative RT-PCR (Figure 4A). In the reproductive tract of male chickens, *TOM1L1* expression increased 5.2- fold (*P*<0.001) in testis at 12weeks and decreased 70% (*P*<0.05) in testes of at 50weeks in male chickens as compared with *TOM1L1* expression in the gonads at E14 (Figure 4B). In addition, TOMIL1 expression was strong in the oviduct of 12-week-old chickens and moderately expressed in ovarian follicles of 12- and 50-week-old female chickens (Figure 5A and 5B). In male chickens, *TOM1L1* mRNA and protein were most abundant in the seminiferous cord of embryonic gonads at E14 and E18, seminiferous tubules of immature testis (12 wks) and spermatogonia of testes at 50 weeks (Figures 6A and 6B).

### Comparative Expression of TTR during Development of Reproductive Organs in both Sexes of Chickens

**Figure 7:**
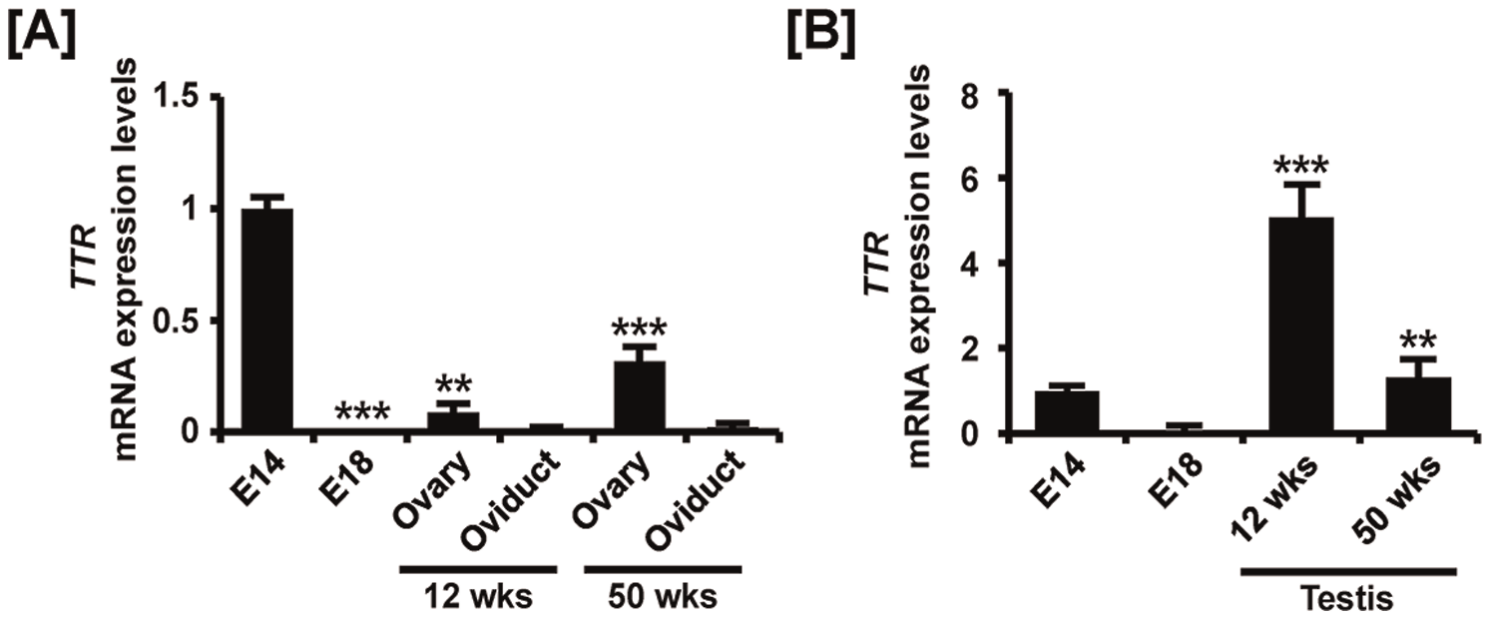
Quantitative analysis of *TTR* mRNA expression in female and male reproductive tracts during their development. Quantitative RT-PCR was conducted using cDNA templates from female (A) and male (B) gonads at embryonic days 14 and 18, 12-week-old ovary and oviduct and 50-week-old ovary and oviduct. The asterisks denote statistically significant differences (*** *p*<0.001 and ** *p*<0.01).

**Figure 8:**
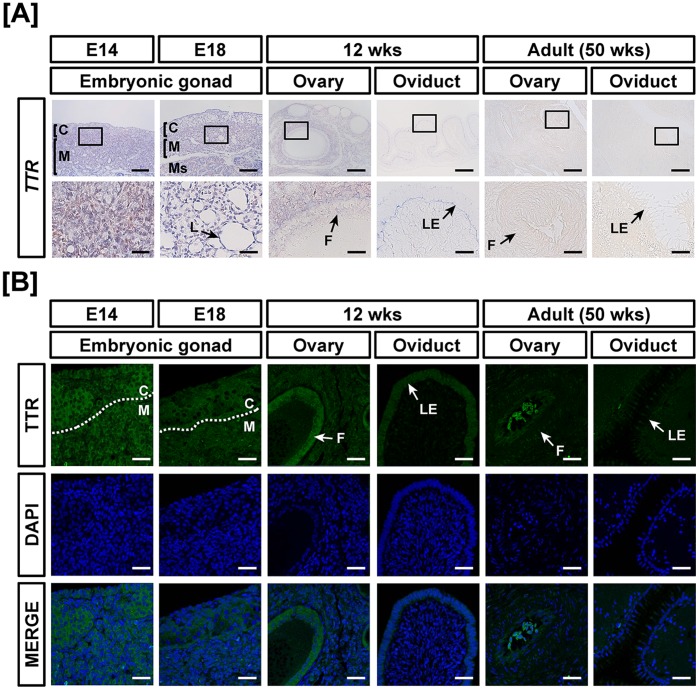
Cell-specific localization of mRNA and protein for TTR in female reproductive tracts during their development. Cell-specific expression of *TTR* mRNA and protein in development of the female reproductive tract was demonstrated by *in situ* hybridization (A) and immunofluorescence analyses (B). Cell nuclei were stained with DAPI (blue). Legend: C, cortex; F, follicle; L, lacunae; LE, luminal epithelium; M, medullar; Ms, mesonephros; S, stroma. Scale bar represents 100 µm and 20 µm for first and second horizontal panels of (A) and 50 µm for (B). See *Materials and Methods* for a complete description of the methods.

**Figure 9:**
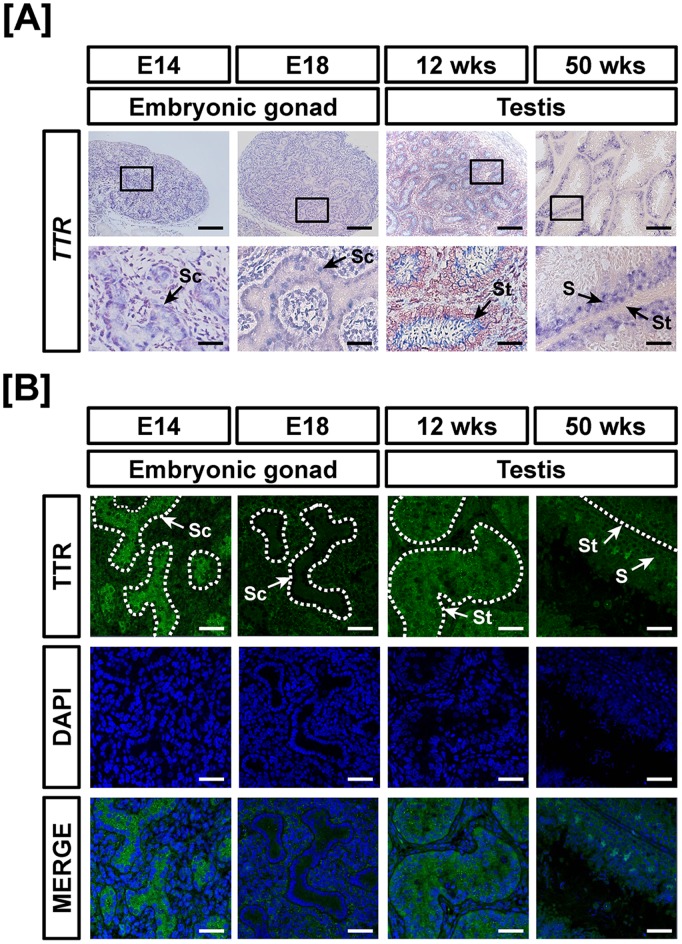
Cell-specific localization of mRNA and protein for TTR in male reproductive tracts during their development. Localization of TTR expression was analyzed in the male reproductive tract of chickens during their development by *in situ* hybridization (A) and immunofluorescence analyses (B). Cell nuclei were stained with DAPI (blue). Legend: S, Sertoli cell; Sc, seminiferous cord; St, seminiferous tubule. Scale bar represents 100 µm and 20 µm for first and second horizontal panels of (A) and 50 µm for (B). See *Materials and Methods* for a complete description of the methods.

Chicken TTR gene expression was demonstrated in the reproductive tract during development of ovaries, oviduct and testes. In females, *TTR* was expressed weakly during development of the ovary and oviduct. The expression levels indicated 0.01- (*P*<0.001), 0.08- (*P*<0.01), 0.02-, 0.3- (*P*<0.001) and 0.02- fold changes in *TTR* mRNA in the embryonic gonads at E18, 12-week-old ovaries and oviducts and 50-week-old ovaries and oviducts as compared with TTR expression in the embryonic gonads at E14 (Figure 7A). Next, *TTR* mRNA was evaluated during testis development in chickens. The results showed that TTR expression decreased 92% in the embryonic gonads at E18 and increased 5.1- (*P*<0.001) and 1.3- (*P*<0.01) fold in testes of 12- and 50-week-old chickens, respectively as compared with expression at E14 (Figure 7B). In accordance with quantitative mRNA expression, cell-specific expression, based on results from *in situ* hybridization and immunofluorescence analyses, revealed that TTR is expressed mainly in the cortex of embryonic gonads, whereas its expression is rarely detected in other tissues of the female reproductive tract (Figure 8A and 8B). Furthermore, TTR was localized predominantly to the seminiferous cords of embryonic gonads (E14), seminiferous tubules of immature testis (12 wks) and Sertoli cells of adult testes (50 wks) as shown in Figures 9A and 9B.

### Comparative Expression of ZEB1 during Development of Reproductive Organs in Female and Male Chickens

**Figure 10:**
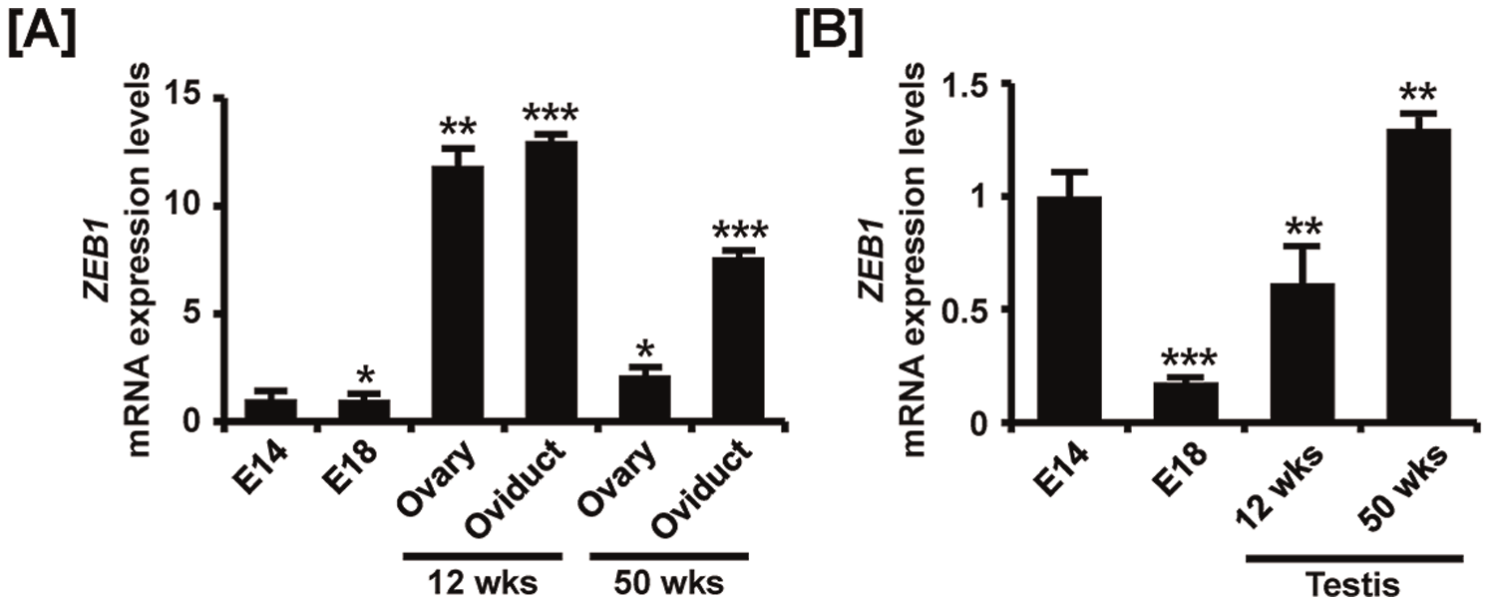
Quantitative analysis of *ZEB1* mRNA expression in female and male reproductive tracts during their development. Quantitative RT-PCR was conducted using cDNA templates from female (A) and male (B) gonads at embryonic days 14 and 18, 12-week-old ovary and oviduct and 50-week-old ovary and oviduct. The asterisks denote statistically significant differences (*** *p*<0.001, ** *p*<0.01 and * *p*<0.05).

**Figure 11:**
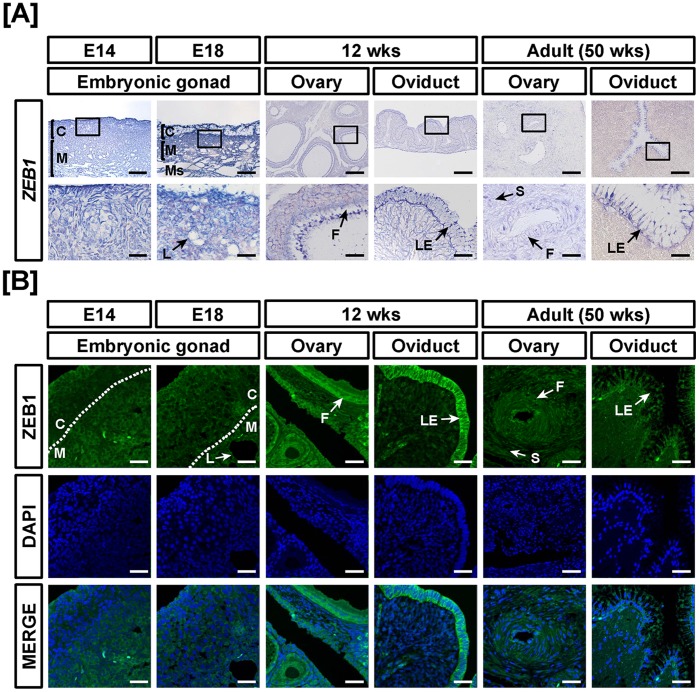
Cell-specific localization of mRNA and protein for ZEB1 in female reproductive tracts during their development. Cell-specific expression of *ZEB1* mRNA and protein in development of the female reproductive tract was demonstrated by *in situ* hybridization (A) and immunofluorescence analyses (B). Cell nuclei were stained with DAPI (blue). Legend: C, cortex; F, follicle; L, lacunae; LE, luminal epithelium; M, medullar; Ms, mesonephros; S, stroma. Scale bar represents 100 µm and 20 µm for first and second horizontal panels of (A) and 50 µm for (B). See *Materials and Methods* for a complete description of the methods.

**Figure 12:**
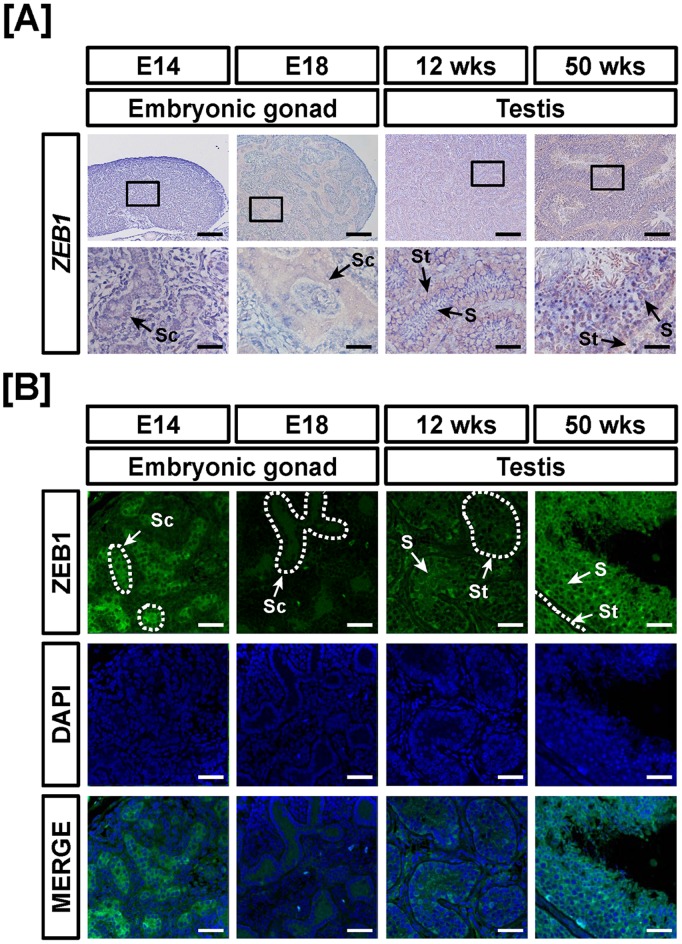
Cell-specific localization of mRNA and protein for ZEB1 in male reproductive tracts during their development. Localization of ZEB1 expression was analyzed in the male reproductive tract of chickens during their development by *in situ* hybridization (A) and immunofluorescence analyses (B). Cell nuclei were stained with DAPI (blue). Legend: S, Sertoli cell; Sc, seminiferous cord; St, seminiferous tubule. Scale bar represents 100 µm and 20 µm for first and second horizontal panels of (A) and 50 µm for (B). See *Materials and Methods* for a complete description of the methods.

Tissue-specific expression of ZEB1 in the female and male reproductive tracts of chickens during development was demonstrated using quantitative RT-PCR, *in situ* hybridization and immunofluorescence analyses. As illustrated in Figure 10A, *ZEB1* mRNA expression increased in the reproductive tracts of 12- and 50-week-old female chickens as compared to embryonic gonads. Its expression increased 11.9- (*P*<0.01), 13- (*P*<0.001), 2.2- (*P*<0.05) and 7.6- (*P*<0.001) fold in 12-week-old ovaries and oviducts and 50-week-old ovaries and oviducts, respectively as compared with *ZEB1* expression in the gonads at E14. In male chickens, ZEB1 expression increased gradually from E18 gonads to adult testes. Expression of *ZEB1* mRNA increased 0.2- (*P*<0.001), 0.6- (*P*<0.01) and 1.3- (*P*<0.01) fold in the gonads at E14 and E18 and testis of 12- and 50-week-old male chickens, respectively as compared with *ZEB1* expression in the gonads at E14 (Figure 10B). *ZEB1* mRNA and protein were highly expressed in luminal epithelium of the oviduct at 12- and 50-weeks of age and in ovarian follicles of ovaries from 12-week-old female chickens (Figure 11A and 11B). In addition, ZEB1 was weakly expressed in the cortex and medullary region of gonads at E14 and E18. In male reproductive organs, *ZEB1* mRNA and protein were localized predominantly to seminiferous cords of E14 gonads and Sertoli cells of testes from 50-week-old male chickens (Figure 12A and 12B). These results suggest that ZEB1 participates in development of both the oviduct and testis in chickens.

### Post-transcriptional Regulation of Genital Ridge Development Regulatory Genes by Chicken microRNAs

**Figure 13:**
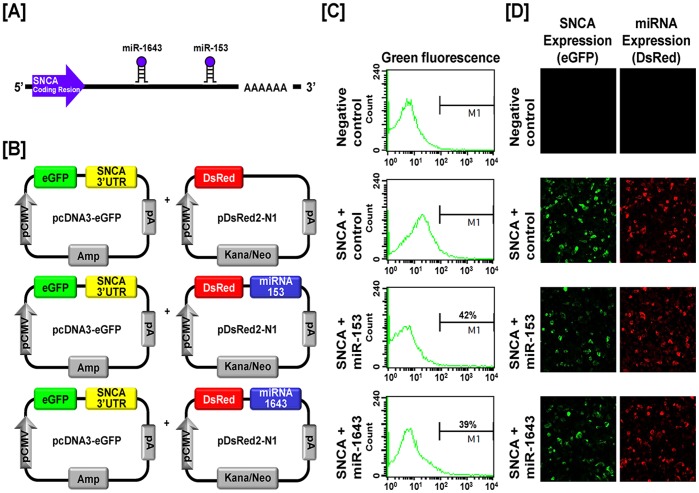
*In vitro* target assay for *miR-153* and *miR-1643* on the SNCA transcript. (A) Diagram showing *miR-153* and *miR-1643* binding sites in SNCA 3′-UTR. (B) Schematic expression of vector maps for eGFP with SNCA 3′-UTR and DsRed with each miRNA. (C and D) The fluorescence signals of GFP and DsRed were detected using FACS (C) and fluorescent microscopy (D) after co-transfection of pcDNA-eGFP-3′-UTR for the *SNCA* transcript and pcDNA-DsRed-miRNA for the *miR-153* and *miR-1643*.

**Figure 14:**
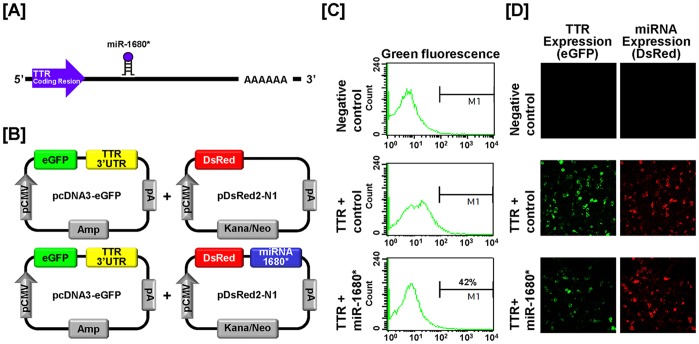
*In vitro* target assay for miR-1680* on the TTR transcript. (A) Diagram showing miR-1680* binding sites in TTR 3′-UTR. (B) Schematic expression of vector maps for eGFP with TTR 3′-UTR and DsRed with each miRNA. (C and D) The fluorescence signals of GFP and DsRed were detected using FACS (C) and fluorescent microscopy (D) after co-transfection of pcDNA-eGFP-3′-UTR for the *TTR* transcript and pcDNA-DsRed-miRNA for miR-1680*.

**Figure 15:**
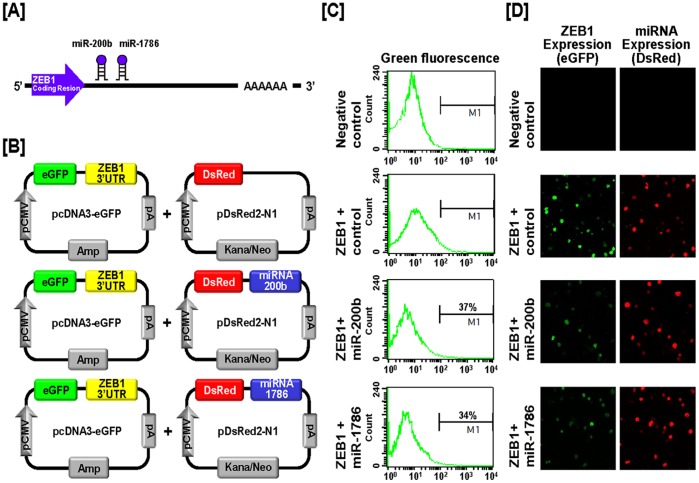
*In vitro* target assay for *miR-200b* and *miR-1786* on the ZEB1 transcript. (A) Diagram showing *miR-200b* and *miR-1786* binding sites in ZEB1 3′-UTR. (B) Schematic expression of vector maps for eGFP with ZEB1 3′-UTR and DsRed with each miRNA. (C and D) The fluorescence signals of GFP and DsRed were detected using FACS (C) and fluorescent microscopy (D) after co-transfection of pcDNA-eGFP-3′-UTR for the *ZEB1* transcript and pcDNA-DsRed-miRNA for the *miR-200b* and *miR-1786*.

We next investigated the possibility that expression of *SNCA*, *TTR* and *ZEB1* is regulated at the post-transcriptional level by microRNAs (miRNAs) using a miRNA target validation assay. In order to find target miRNAs and their binding sites within the 3′-UTR of *SNCA*, *TTR* and *ZEB1* genes, the miRNA target prediction database (miRDB: http://mirdb.org/miRDB/) was used. It revealed several putative binding sites for miRNAs including *miR-153* and *miR-1643* for SNCA, miR-1680* for TTR and *miR-200b* and *miR-1786* for ZEB1 (Figures 13, 14 and 15). However, no specific target miRNA was detected for TOM1L1. Thus, we determined if these specific miRNAs influence expression of SNCA, TTR and ZEB1 via their 3′-UTR. A fragment of each 3′-UTR with binding sites for the miRNAs was cloned downstream of the green fluorescent protein (GFP) reading frame, thereby creating a fluorescent reporter for function of the 3′-UTR region (Figure 13B, 14B and 15B). After co-transfection of eGFP-3′-UTR and DsRed-miRNA, analyses for intensity of GFP expression and percentage of GFP-expressing cells were conducted using FACS and fluorescence microscopy. In the presence of *miR-153* and *miR-1643* decreased the intensity and percentage of GFP-SNCA-expressing cells 58% and 61% (Figure 13). In addition, miR-1680* decreased the intensity and percentage of cells expressing TTR by 58% (Figure 14). Furthermore, *miR-200b* and *miR-1786* decreased the intensity and percentage of GFP-ZEB1-expressing-cells by 63% and 66%, respectively (Figure 15). These results indicate that specific miRNAs associated with target transcripts may be involved in development of reproductive organs in chickens and regulate their expression at the post-transcriptional level during morphogenesis of the ovary, oviduct and testis.

## Discussion

Results of the current study revealed differential temporal and spatial expression patterns for key genes, SNCA, TOM1L1, TTR and ZEB1 that are important for development and differentiation of chicken reproductive tract in both sexes. In addition, the results indicate that among the four selected genes, expression of SNCA, TTR and ZEB1 is post-transcriptionally regulated via specific miRNAs binding directly the 3′-UTR of these target genes. These results support our hypothesis that molecular patterning of the reproductive system is affected by prominent transcripts crucial for development of female and male reproductive organs in chickens.

The chicken is a firmly entrenched animal model for research in embryology and reproductive developmental biology, but little is known about regulatory genes that control development of the reproductive tract in female and male chickens. In our previous study (Lim and Song, 2014, in submission), we reported novel genes and hormonal regulation of gonad morphogenesis in chicken embryos. To demonstrate the differential patterns of expression of mRNAs and proteins of SNCA, TOM1L1, TTR and ZEB1 during development and differentiation of germ cells and primordial tissues to mature reproductive organs, we performed quantitative RT-PCR, *in situ* hybridization and immunofluorescence analyses.

SNCA belongs to a family of small and highly conserved proteins in vertebrates including alpha-, beta- and gamma-synuclein. The SNCA gene has 7 exons (5 protein-coding) and is expressed mainly in the brain, particularly in the hippocampus, caudate nucleus, amygdala, substantia nigra and thalamus in adult humans [19]. The SCNA gene has been specifically related to several neurodegenerative diseases such as Parkinson’s disease via three point mutations (A53T, A30P and E46K) [20], [21] and Alzheimer’s disease through accumulation of the gene product in humans [22]. In addition, testosterone increases *SNCA* mRNA expression in the brain of chipping sparrows to affect their song system [23]. In the chicken, there is expression of SNCA in the majority of neurons in brain and spinal cord during embryogenesis [24]. However, there are no published results on SNCA and development of reproductive organs of any animal or human model. We reported that SNCA is expressed in both male and female embryonic gonads in chickens. Therefore, we investigated expression of SNCA during development of the reproductive organs from embryonic gonads to adult male and female reproductive tracts. SNCA was detected predominantly in ovary and testis of 12-week-old chickens which suggests that SNCA might have an important role in morphogenesis of ovary and testis.

TOM1L1 is also known as Src-activating and signaling molecule (SRCASM). This gene is an activator and substrate for Src family tyrosine kinases (SFKs) that include nine members that have significant roles in mitogenesis and morphological alterations via induction of growth factors [25]. TOM1L1 is tyrosine-phosphorylated in response to EGFR ligand as a SFK substrate downstream of EGFR. Increased expression of TOM1L1 activates endogenous SFKs preferably for phosphorylating Fyn and Src. Therefore, TOM1L1 links with EGFR and SFK-dependent signaling in differentiation of keratinocytes [26], [27]. In addition, TOM1L1 has a role as a regulatory adaptor bridging activated EGFR in endocytosis by EGF stimulus [28]. In the present study, we determined that TOM1L1 was expressed strongly in luminal epithelium of the immature oviduct and follicles of adult ovaries in female chickens and in testes of 12-week-old male chickens. These results show that TOM1L1 likely has a role in regulating development of the immature oviduct, ovarian folliculogenesis and seminiferous tubules in chickens.

TTR (also called prealbumin) is one of the transporters of thyroid hormones and cooperates with retinol-binding protein (RBP) and vitamin A (retinol). TTR directly binds the thyroid hormones (T_3_ and T_4_) in the central channel constituted by tetrameric assembly of the monomers [29], [30], [31], and it indirectly provides vitamin A as retinol bound to RBP [32]. TTR has a well-established role in regulating spermatogenesis through effects on retinol metabolism in the adult testis of rats. Circulating retinol binds to a complex of RBP and TTR which is present in the peritubular cells associated with intracellular CRBP which has a high affinity for binding retinol [33]. The peritubular cells secrete retinol as a complex form to the Sertoli cells that oxidize retinol into retinoic acid which stimulates differentiation of germ cells [34]. Therefore, TTR assists in development of germ cells within the developing seminiferous tubules. In females, it is not known if TTR regulates oogenesis or embryogenesis. However, thyroid hormones transported by TTR from serum into the oocyte play a crucial role in embryogenesis in various species, especially as it is accumulates in the yolk of oocytes during oogenesis in chickens [35]. In our study, TTR was highly expressed during testes development, predominantly in the seminiferous tubules of immature testes. Otherwise, in the female reproductive tract, expression of TTR was weak in immature and mature ovaries. These results indicate that the TTR gene might have an important role in development and maturation of the postnatal testis in chickens.

ZEB1 (also known as EF1, TCF8, AREB6, Nil-2-a) is a transcription factor binding to DNA via two zinc finger clusters, one at the N-terminus and one at the C-terminus, and it can modulate transcription of target genes by binding directly to 5′-CACCT sequences in their promoter regions [36]. ZEB1 plays an important role in development, cell proliferation, differentiation, migration and reproduction [37], [38]. In addition, ZEB1 protein induces cell migration during development and cancer progression by repressing expression of E-cadherin in epithelial cells [39], [40], [41], [42]. ZEB1 is regulated by steroid hormones, estrogen [43], progesterone [44] and androgen [45]. In chickens, estrogen induces proliferation and differentiation of tubular gland cells associated with production of egg white proteins and stimulates ZEB1 expression leading to activation of transcription of downstream targets in the chick oviduct [43], [46]. In this study, ZEB1 was highly expressed in both the immature and mature female reproductive tract as compared to the embryonic gonads suggesting a key role in development of the oviduct of adult female chickens. Moreover, in male chickens, ZEB1 expression increased gradually with progressive development of the testes from an E18 gonad to an adult testis. Thus, ZEB1 may play a crucial role in egg production through effects on development of the oviduct, as well as all stages of development of the testis in male chickens.

Based on results from validation of gene expression during development of chicken reproductive organs, we next investigated whether target genes undergo post-transcriptional regulation by specific microRNAs. MicroRNAs (miRNAs) are small non-coding single stranded RNAs of 18–23 nucleotides that play a role as post-transcriptional regulators and transformers of cell fate through modulation of target-mRNA translation in various cells and tissues. In other words, miRNAs have crucial regulatory effects in a variety of biological events including growth, development, differentiation and control of cell cycle by modulating gene expression [47], [48], [49]. For example, expression of miRNAs during gonadal development in chickens and mammals has been reported [50], [51], [52]. In addition, several miRNAs regulate mechanisms required for development and differentiation of the oviduct and ovarian cancer in female chickens [14], [16], [53], [54]. Moreover, *miR-34c* down-regulates genes related to germ cell differentiation and its expression was detected mainly in the later stages of meiosis in spermatogenesis in chickens [55]. Based on previous reports, miRNAs might play a role during morphogenesis of the ovary, oviduct and testis in chickens. However, few miRNAs have been investigated with respect to their regulation of target genes and mechanisms whereby they act remain unknown. In this study, we performed an *in vitro* target assay of miRNAs to determine if *SNCA*, *TTR* and *ZEB1* transcripts are regulated at the post-transcriptional level by target miRNAs. As illustrated in Figures 13, 14, and 15, specific target miRNAs of chickens attenuate intensity of GFP-*SNCA*, -*TTR* and -*ZEB1* expressing cells. These results indicate that at least one to two miRNAs directly bind to the developmental-regulatory genes of reproductive organs and post-transcriptionally regulate their expression during development of the male and female reproductive tracts of chickens.

In conclusion, our results provide evidence for temporal and spatial expression of five genes that influence development of reproductive organs of chickens from the embryonic stage to the immature and mature stages of development. Expression of SNCA, TTR and ZEB1 are modulated via post-transcriptional regulation by specific target miRNAs which warrant further study. These results suggest roles for four important genes that likely regulate development of reproductive organs in chickens.

## Materials and Methods

### Experimental Animals and Animal Care

The experimental use of chickens for this study was approved by the Animal Care and Use Committee of Korea University. All chickens were exposed to a light regimen of 15 h light and 9 h dark with *ad libitum* access to feed and water, and subjected to standard poultry husbandry guidelines.

### Tissue Samples

The left and right gonads were collected separately from the mesonephric kidney of chicken embryos at E14 and E18 in a 1.5 ml tube containing diethylpyrocarbonate treated PBS (DEPC-PBS). Then we centrifuged the sample at 1,080 x g for 5 min to allow collection of each gonad from the bottom of the tubes. After removal of the DEPC-PBS, the gonads were stored at −80°C until RNA was extracted. Also we collected whole embryos and fixed them in freshly prepared 4% paraformaldehyde in PBS (pH 7.4). Tissue samples were collected from ovary, oviduct and testis of 12- and 50-week-old females (n = 4) and males (n = 4). The collected samples were either stored at −80°C until RNA was extracted or fixed immediately upon collection in freshly prepared 4% paraformaldehyde in PBS (pH 7.4). After 24 h, the samples fixed in 4% paraformaldehyde were changed to 70% ethanol for 24 h and then dehydrated in a graded series of increasing concentrations of ethanol. Embryos were then incubated in xylene for 3h and embedded in Paraplast-Plus. Paraffin-embedded tissues were sectioned at 5 µm.

### RNA Isolation

Total cellular RNA was isolated from frozen tissues using Trizol reagent according to manufacturer’s recommendations. The quantity and quality of total RNA was determined by spectrometry and denaturing agarose gel electrophoresis, respectively.

### Quantitative RT-PCR Analysis

Total RNA was extracted from gonads on embryonic day 14 and 18 from both sexes and ovaries, oviducts and testes from 12- and 50-week-old females and males using TRIzol and purified using an RNeasy Mini Kit. Complementary DNA was synthesized using a Superscript III First-Strand Synthesis System. Gene expression levels were measured using SYBR Green and a StepOnePlus Real-Time PCR System. The *glyceraldehydes 3-phosphate dehydrogenase (GAPDH)* gene was analyzed simultaneously as a control and used for normalization of data. *GAPDH* expression is most stable among other housekeeping genes and it is used commonly for normalizing for variations in loading. Each target gene and *GAPDH* were analyzed in triplicate. Using the standard curve method, we determined expression of the examined genes using the standard curves and Ct values, and normalized them using *GAPDH* expression. The PCR conditions were 95°C for 3 min, followed by 40 cycles at 95°C for 20 sec, 60°C for 40 sec, and 72°C for 1 min using a melting curve program (increasing the temperature from 55°C to 95°C at 0.5°C per 10 sec) and continuous fluorescence measurement. ROX dye was used as a negative control for the fluorescence measurements. Sequence-specific products were identified by generating a melting curve in which the Ct value represented the cycle number at which a fluorescent signal was statistically greater than background, and relative gene expression was quantified using the 2^–ΔΔ^Ct method [56]. For the control, the relative quantification of gene expression was normalized to the Ct value for the control oviduct. Information on the primer sets was provided previously (Lim and Song, 2014, in submission).

### In Situ Hybridization Analysis

For hybridization probes, PCR products were generated from cDNA with the primers used for RT-PCR analysis. The products were extracted from the gel and cloned into TOPO TA cloning vector. After verification of the sequences, plasmids containing gene sequences were linearized and transcribed using a DIG RNA labeling kit with T7 or SP6 polymerase. Information on the probes has been published (Lim and Song, 2014, in submission). Tissues were collected and fixed in freshly prepared 4% paraformaldehyde, embedded in paraffin and sectioned at 5 µm on APES-treated (silanized) slides. The sections were then deparaffinized in xylene and rehydrated to diethylpyrocarbonate (DEPC)-treated water through a graded series of alcohol. The sections were treated with 1% Triton X-100 in PBS for 20 min and washed two times in DEPC-treated PBS. After washing in DEPC-treated PBS, the sections were digested with 5 µg/ml Proteinase K in TE buffer (100 mM Tris-HCl, 50 mM EDTA, pH 8.0) at 37°C. After post-fixation in 4% paraformaldehyde, sections were incubated twice for 5 min each in DEPC-treated PBS and incubated in TEA buffer (0.1M triethanolamine) containing 0.25% (v/v) acetic anhydride. The sections were incubated in a prehybridization mixture containing 50% formamide and 4X standard saline citrate (SSC) for at least 10 min at room temperature. After prehybridization, the sections were incubated overnight at 42°C in a humidified chamber in a hybridization mixture containing 40% formamide, 4X SSC, 10% dextran sulfate sodium salt, 10mM DTT, 1 mg/ml yeast tRNA, 1mg/ml salmon sperm DNA, 0.02% Ficoll, 0.02% polyvinylpyrrolidone, 0.2mg/ml RNase-free bovine serum albumin and denatured DIG-labeled cRNA probe. After hybridization, sections were washed for 15 min in 2X SSC at 37°C, 15min in 1X SSC at 37°C, 30 min in NTE buffer (10mM Tris, 500mM NaCl and 1mM EDTA) at 37°C and 30 min in 0.1X SSC at 37°C. After blocking with 2% normal sheep serum, the sections were incubated overnight with sheep anti-DIG antibody conjugated to alkaline phosphatase. The signal was visualized following exposure to a solution containing 0.4 mM 5-bromo-4-chloro-3-indolyl phosphate, 0.4 mM nitroblue tetrazolium, and 2 mM levamisole.

### Immunofluorescence Analysis

The localization of four proteins in the reproductive tract of both sexes during their development was evaluated by immunofluorescence (IF) using anti-human SNCA polyclonal antibody (ab21975), anti-human TOM1L1 polyclonal antibody (ab126972), anti-human TTR polyclonal antibody (ab9015) and anti-human ZEB1 polyclonal antibody (ab81972). Antigen retrieval was performed using boiling 10mM sodium citrate buffer pH 6.0 for 10 min after which the slides were cooled on the bench top for 20 min. After antigen retrieval the slides were washed three times in 1X PBS for 5 min. Slides were incubated in blocking buffer (10% normal serum from the same species as the secondary antibody in 1X PBS) for 1 h. After the blocking solution was aspirated, slides wereincubated overnight at 4°C with primary antibody. The slides were then rinsed three times in 1X PBS for 5 min each. Slides were then incubated with Alexa Fluor 488 rabbit anti-goat IgG secondary antibody for ZEB1, goat anti-rabbit IgG secondary antibody for TOM1L1 and donkey anti-sheep IgG secondary antibody for SNCA and TTR at a 1∶200 dilution for 1 h at room temperature in the dark. Slides were then washed and overlaid with Prolong Gold Antifade with DAPI. For primary antibody, images were captured using a Zeiss confocal microscope LSM710 fitted with a digital microscope camera AxioCam using Zen 2009 software.

### MicroRNA Target Validation Assay

The 3′-UTR of SNCA, TTR and ZEB1 were cloned and confirmed by sequencing. Each 3′-UTR was subcloned between the eGFP gene and the bovine growth hormone (bGH) poly-A tail in pcDNA3eGFP to generate the eGFP-miRNA target 3′-UTR (pcDNA-eGFP-3′UTR) fusion constructs. For the dual fluorescence reporter assay, the fusion constructs containing the DsRed gene and target miRNAs were designed to be co-expressed under control of the CMV promoter (pcDNA-DsRed-miRNA). The pcDNA-eGFP-3′UTR and pcDNA-DsRed-miRNA (4µg) were co-transfected into 293FT cells using the calcium phosphate method. When the DsRed-miRNA is expressed and binds to the target site of the 3′-UTR downstream of the GFP transcript, green fluorescence intensity decreases due to degradation of the GFP transcript. At 48 h post-transfection, dual fluorescence was detected by fluorescence microscopy and calculated by FACSCalibur flow cytometry. For flow cytometry, the cells were fixed in freshly prepared 4% paraformaldehyde and analyzed using FlowJo software.

### Statistical Analyses

All quantitative data were subjected to analysis of variance (ANOVA) according to the general linear model (PROC-GLM) of the SAS program. All tests of significance were performed using the appropriate error terms according to the expectation of the mean square for error. Data are presented as mean ± SEM unless otherwise stated. Differences with a probability value of *P*<0.05 were considered statistically significant.
